# The Hospital. Nursing Section

**Published:** 1901-12-21

**Authors:** 


### The Hospital.

### Nursing Section.

Contributions for this Section of "The Hospital" should be addressed to the Editor, "The Hospital" Nursing Section, 28 & 29 Southampton Street, Strand, London, W.C.

No. 795.—Vol. XXXI. *SATURDAY, DECEMBER* 21, 1901.

### Notes on News from the Nursing World.

### THE KING AND THE RED CROSS DECORATIONS.

Thirteen ladies received the investiture of the Red Cross at the hands of the King on Tuesday at St. James's Palace as follows: — Lady Chesham (Yeomanry Hospital); Lady Sarah Wilson (Mafeking); Hon. Mrs. Agnes Mary Goldmann (Johannesburg); Nursing Sister Edith McCall Anderson, A.N.S.R. (Bloemfontein); Miss Mary C. Fisher, A.N.S.R. (Yeomanry Hospital); Mrs. Gunning, wife of the late Colonel H. Gunning, King's Royal Rifle Corps (Natal and Maritzburg); Miss Amy Knaggs, A.N.S R. (Bloemfontein); Mrs. Eugenie Ludlow, A N.S.R. (distinguished service at the siege of Ladysmith); Miss Annie Maude MacDonnell (now lady superintendent, Richmond, Whitworth, and Hardwicke Irish Hospital), A.N.S.R.; Miss Edith Pretty, A.N.S.R. (Portland Hospital); Miss Jane Elizabeth Skillman, A.N.S.R.; Miss Annie Blanche Trew, A.N.S.R.; and Miss J. H. N. Williamson, New Zealand Nursing Service.

### THE QUEEN AND THE VICTORIAN ORDER.

Intense satisfaction has been given in Canada by the announcement that the Queen, in a telegram to the Countess of Minto, wife of the Governor-General, has consented, "with great pleasure," to become patroness of the Victorian Order of Nurses and of the Ottawa hospitals. The interest which her Majesty takes in the nursing movement has always extended to our empire beyond the seas, but the association of her name with the Victorian Order is a practical demonstration whose value cannot be measured in words.

### ROYAL BRITISH NURSES' SETTLEMENT FUND.

Princess Christian, as President of the Royal British Nurses' Association, will not only open the sale in aid of the Royal British Nurses' Settlement Fund, which is to take place at Lord Brassey's house in Park Lane on February 6 and 7, but she has also promised to preside herself at one of the stalls. The Fund is intended to provide nurses with comfortable quarters in old age. Over £800 has already been subscribed, including the latest donation of £110 from the lady superintendent of the Cardiff Infirmary.

### THE WAR NURSES.

The *Aurania* arrived at Southampton from South Africa on Wednesday the 11th, when the following nursing sisters disembarked: — A. E. Andre, A.N.S.R., E. Newbolt, A.N.S.R., and A. M. Chatfield, Colonial Nurse. All require one month's leave before returning to South Africa. We learn with regret that Nursing Sister Anne Blackley Denten is seriously ill at Mafeking, from a disease, not diagnosed on December 11th. Sisters M. T. Enright and B. Roberts have, happily, been discharged from hospital.

### THE SOUTH AFRICAN CONSTABULARY.

We have already stated that there are no more vacancies in the nursing establishment of the South African Constabulary, and we now learn from the principal medical officer at Johannesburg that so many names are down for future vacancies that an indefinite time must elapse before any more are required. The nursing service itself will not, we understand, be organised on definite lines until peace conditions prevail. At present the Constabulary have to keep pace with the fighting columns scattered all over the Transvaal and the Orange River Colony, and none of their permanent hospitals or barracks are even commenced yet.

### NURSES FOR THE COLONIES.

The proceedings last week at the meeting of the Scottish branch of the Colonial Nursing Association at Glasgow were extremely interesting. After the Lord Provost, Lord Balfour of Burleigh, and Lord Glasgow had urged the claims of the association to the support of the Scottish people, Lord Lamington, who has just come back from Queensland, testified from his personal experience as Governor of that colony, that the aid which might be given to the white population in New Guinea by nurses going out there was inconceivable. Such aid he regarded as not only directly practical, but he also believed that it was at the same time the highest form of civilisation. General Sir Archibald Hunter then referred in terms of enthusiasm to the services rendered by nurses in Ladysmith, and declared his conviction "that the greatest cause of mortality in disease was the want of good nursing, and the greatest cause of the wonderful recoveries that were made was due to the fact of having a good, attentive, skilful woman nurse to smooth the pillow, to give the patient his beef tea and his medicine at the proper time." The value of the work of nurses in Australia and South Africa having been attested on the highest authority, Colonel Man Stuart told the audience how immensely it is appreciated in West Africa. "He was in command of the lines of communication under Sir Jas. Willcocks in the late Ashantee campaign. There were three or four ladies there who took charge of the nursing, and, like all men who served under Sir Jas. Willcocks, he had the greatest love and admiration for these ladies. He spoke feelingly on this subject, because he had been sick unto death and had been nursed back to health by matron Ward and nurse Nevill, and able to return to duty and see the campaign through." Such testimony as this in respect to nursing in three different parts of Greater Britain is at once an irresistible reason for strengthening the hands and enlarging the borders of the Colonial Nursing Association, and a crushing refutation of the mendacious assertion that the services rendered by those who are engaged in the work are of small account.

### MORE NURSES FOR THE CONCENTRATION CAMPS.

We understand that a further number of nurses are leaving England in the *Simla* to undertake duty in the Concentration Camps in South Africa. They include Miss Bertha Sutcliffe and Miss Elizabeth S. Rabarts. Miss Sutcliffe was trained at the Hospital for Women and Children at Leeds. She has since had charge of the convalescent ward of the Samaritan Free Hospital for Women and Children ; been charge nurse of the female wards of Croydon General Hospital ; and night nurse at the Holborn Union Infirmary, Upper Holloway. Miss Sutcliffe had the honour of receiving a testimonial from Miss Florence Nightingale. Miss E. S. Rabarts was trained at St. Marylebone Infirmary, where she remained for four years. She was then three years sister at the Southwark Infirmary, East Dulwich ; and for four years night superintendent at the Brentford Union Infirmary, Isleworth, where she held the post of assistant matron for the last year and three months.

### CHRISTMAS AT THE HOSPITALS.

Owing to the prevalence of small-pox in London it is probable that the Christmas festivities at King's College Hospital will be of a quiet character, and though every effort will be made by the staff to give a pleasant time to the patients, no public entertainment will be possible, as patients' friends are not being admitted to the wards at present.—As most of the patients at the Bolingbroke Hospital, Wandsworth Common, are adults there will be no Christmas tree, but there will be plenty of quiet enjoyment for both patients and staff. Hanging on the wall in the room of the matron is the original drawing of the nurses entertaining their visitors, and the patients with their friends, which was reproduced in the *Daily Graphic* a year or so ago, and the programme this Christmas will be on much the same lines as hitherto.

### CHRISTMAS FOR THE CHILDREN.

There will be Christmas trees in all the wards at Great Ormond Street Hospital, and on Tuesday, 31st, New Year's Eve, a Christmas entertainment will be given from 3 to 6. Flowers, plants, and fairy lamps will decorate the wards, and everyone is prepared to work hard to make the time a happy one for the little ones. There will be no entertainments in the out-patients' department this year.—At the Alexandra Hospital for Hip Disease signs of the coming season were visible in the hall when our representative called, a week before Christmas Eve ; and though the matron regrets the fact that there is very little money to do it with, it is plain that she means to do the best she can for the little patients. On Monday and Tuesday in Christmas week there will be Christmas trees in the afternoon ; on Christmay Day all the children will have their toys, and a grand surprise is being prepared for them by the nurses, who are going to give some tableaux vivants. The scenes to be presented are just those the children love—" Little Red Riding Hood," " Cinderella," etc. ; while a novel item on the programme is "The Seasons," and the tableaux performers will visit the wards in turn, so that all the children may see them. During the day the chaplain will tell the story of Christmas. About the middle of January will be the great day, however, when some 200 or 300 guests are invited to an entertainment.

### THE DUDLEY WORKHOUSE SCANDAL.

The inquiry, under the auspices of Mr. E. B. Wethered, Local Government Board Inspector, relative to the charges preferred against Miss M. M. B. Newbury, superintendent nurse at the Dudley Workhouse Infirmary, and also to charges preferred by Miss Newbury against certain officers of the Dudley Workhouse, which commenced on the 26th of November, has now been concluded. It occupied, at intervals, no less than seven days, and closed on Friday, the 13th. But it may yet be some time before the decision of the Local Government Board is given. We can only therefore say at present that the evidence, which was of a most conflicting character, proves beyond doubt that there was a necessity for investigation. The result of the inquiry must, in any case, be of considerable importance, and will be awaited with interest far beyond the limits of Dudley or the Midland counties.

### SEVEN SUPERINTENDENTS IN TWO YEARS.

The constant changes in the nursing staff of the Shrewsbury Workhouse Infirmary seems to perplex some of the members of the Atcham Board of Guardians, and at the last meeting it was proposed that the nurses should be interviewed by the Officers' Committee. The master declared that whatever might be the explanation of the last nurse leaving, it was not due to any friction between her and himself or the matron, and this statement was not at the time called in question. But, as Dr. Cureton said that there had been, he believed, seven superintendents during the last two years, an inquiry into the cause of the changes is certainly called for. The chairman observed that before they appointed a superintendent the guardians never had any trouble at all with the nurses, a state of affairs which may have been due to the absence of discipline. The whole of the seven superintendents can hardly have been cantankerous or over-bearing.

### A MATRON'S ACTION FOR LIBEL.

One of the outcomes of the dismissal of Miss Martin from the post of matron of the Belper Isolation Hospital, in the summer of 1900, was a libel action against the medical superintendent, which was tried at the last Assizes at Leeds before the Lord Chief Justice and a special jury, with the result that the jury disagreed. A new trial, which lasted three days, was concluded at the Leeds Assizes on Saturday. Miss Martin claimed damages against Dr. Allen on the ground that a letter he wrote to the hospital committee led to her dismissal. In this letter he described her as "absolutely unfitted for the responsible position in which she was placed," and said that " she appears to think that this building is erected for her sole use and benefit, and is a convenient centre for friendly visitation and dissemination of idle gossip," that "petty annoyance by her made life miserable," and that he had received " more insolence from her in six months than in all his experience with nurses for a quarter of a century." The defence was that the letter in which these and other allegations were made was privileged, and that the allegations were substantially correct. A number of witnesses were examined, and the evidence elicited from Mr. Justice Grantham the remark that he congratulated the jury on not having been among the happy family in the Isolation Hospital. But in the end a verdict for the plaintiff was awarded with damages of £100. The amount is not in the circumstances excessive, but Miss Martin will probably value more than the pecuniary compensation she receives the vindication of her professional reputation.

### A SUPERINTENDENT AND HER SALARY.

The nursing question is again troubling the Devonport Board of Guardians, and Dr. Rowe, the medical officer, told the board the other day that it was likely to become increasingly difficult. Dr. Rowe was able to cite incontestible evidence in support of his assertion. The superintendent nurse is leaving the infirmary. When she was appointed she was the only applicant. Now, in view of her departure, the Board have advertised again, and the solitary reply was from a person described as "half-qualified." Apparently in this instance the vacancy is occurring simply because the infirmary committee would not make the small addition to the nurse's salary desired by her. She is admittedly a competent superintendent, midwife, and nurse, and the Board have very wisely decided to make an effort to keep her, and referred the matter back to the committee. As Dr. Rowe says, there is no lack of candidates for the position of nurse in general and military hospitals, but in many infirmaries nurses cannot be obtained at all.

### THE RESIGNATIONS AT HALIFAX.

An official report of the resignations of Miss Wilkie, matron of the Workhouse Hospital, Halifax, Miss Bolton, assistant matron, and charge nurses Margaret Gordon, Florence Vernon, Mary Whitehouse, Isabella Davy, and Edith Wingrave, has now been given, but as many of the members of the Halifax Board of Guardians confess themselves still in doubt as to the cause of the action taken, it may be imagined that no satisfactory explanation has been forthcoming. Miss Wilkie has obtained an appointment under the Government in South Africa, which proves that her credentials are unimpeachable. But it is not pretended that she relinquished the post of matron at Halifax merely in order to accept it. Nor has any successful attempt been made by the Board to throw light on the fact that concurrently with her resignation those of the assistant matron and five charge nurses were announced. The chairman of the Infirmary and Hospital Committee avers that they are leaving simply because Miss Wilkie is leaving. But we agree with a guardian who contended that the numerous and simultaneous resignations show that there is something that requires investigation. Miss Wilkie cannot be blamed for her reticence. It is clear that she has always been on the best of terms with her staff, and the only information she has afforded to anyone is that "she does not see eye to eye with the committee." This points to the conclusion that there have really been important differences of opinion, and we regret, in the interests of the Hospital itself, that it has been decided to let the matter drop without further inquiry. For what guarantee is there that the next matron will see eye to eye with the committee? If not, will she also go and take part of her staff with her? The issue should be threshed out before the appointment of Miss Wilkie's successor.

### FULHAM AND HAMMERSMITH DISTRICT NURSES.

The annual social gathering of the Fulham and Hammersmith District Nursing Association took place last week at Carnforth Lodge, when the collecting books were brought in. These were received by Mr. Hayes Fisher, M.P. for Fulham, who read out the list of collectors and amounts; the total sum received was £80. In speaking of the work of the Association, Mr. Fisher said it had been computed that for every 9,000 of the population of a district there should be one nurse; and if this were realised in Fulham and Hammersmith, there should be twenty-seven nurses instead of eight.

### THE STAFF OF THE METROPOLITAN ASYLUMS BOARD.

At the usual fortnightly meeting of the Metropolitan Asylums Board, on Saturday, a letter was read from the Local Government Board assenting to the appointment of Miss M. Jones as temporary matron at the Gore Farm Hospital, and approving the salary and other emoluments which it is proposed to assign to her. On the recommendation of the finance committee, it was decided that a superannuation allowance of £22 19s. per annum be paid to Miss Ellen Cain, late charge nurse at the Western Hospital. The Board confirmed the appointments of Miss S. A. Villiers and Miss A. Thomas as matrons of the Fountain and Park Hospitals respectively, and agreed that, subject to the approval of the Local Government Board, they should be paid salaries at the rate of £100 per annum each, rising by £5 annually to £150, together with the emoluments of furnished apartments, rations, attendance, coal, light, and washing.

### DISTRICT NURSING AT REDRUTH.

The Redruth District Nursing Committee are again able to give a satisfactory record of nursing work done during the year, and also to report that subscriptions keep up well. The credit balance from last year and receipts this year, including offerings for services rendered by one nurse, were altogether £111, and the expenditure £73, leaving a credit balance of nearly £40 to be carried forward. The nurse, who commenced work early in December last year, has been kept fully engaged, 3,395 visits to 97 patients suffering from various sicknesses and accidents, some very serious, having been paid during her eleven months' work. Mrs. Langon, hon. secretary and treasurer for the past seven years, has consented to continue these duties for another year.

### SHORT ITEMS.

The Guardians of the Steyning Union have agreed to subscribe £15 15s. annually to the Brighton District Nursing Association in connection with Queen Victoria's Jubilee Institute.—Mr. Ismay has given £150 towards tarting a Queen's Nurse for the Alcester district, as a memorial to the late Lady Margaret Ismay, who always took great interest in the nursing of the poor.—Four nurses who were instructed at the Brentford Union Infirmary, Isleworth, successfully passed the examination held by the Incorporated Society of Trained Masseuses on November 26th and 27th, and have obtained the Society's certificate.—Miss Bryant, of the English Nurses' Institute, San Remo, asks us to say that it is impossible to answer letters from nurses unless 2½d. in stamps are enclosed for reply, and that the postage to Italy is 2½d., not a penny.

### Lectures to Nurses on Medicine and Medical Nursing.

By Norman Dalton, M.D. London, F.R.C.P., Physician to King's College Hospital.

### LECTURE XXVI.—APOPLEXY.

The word "apoplexy" means a stroke or knock-down blow, and from the fact that in many cases of cerebral hæmorrhage the patient falls as if knocked down, apoplexy has come to mean much the same thing as cerebral hæmorrhage. Nurses, and even students, are apt to confuse apoplexy with "hemiplegia.' Hemiplegia means paralysis of one side, and it is a common result of cerebral hæmorrhage, so that apoplexy and hemiplegia are most frequently found together; but apoplexy is the stroke, or fit, or attack, while hemiplegia is the resulting paralysis and usually persists after the apoplexy has passed off.

Cerebral hæmorrhage results from a diseased condition of the arteries, which makes them brittle. In people whose arteries are affected in this way the radial artery can be felt to have thick rigid walls, and the temporal artery stands out as a twisted cord. Such arteries are natural to old people, and consequently cerebral hæmorrhage is usually met with in the old; but if from any cause the arteries degenerate at an earlier age than usual their rupture is even more likely to occur than in old people, because the heart and muscles of middle-aged people may be strong, and when they act vigorously, they throw a great strain upon the diseased arteries and frequently cause them to break, whereas the feeble heart and muscles of old people cannot produce more strain than the weak arteries can bear. Middle-aged people, with diseased arteries, should therefore be warned against vigorous exertion. Persons suffering from chronic Bright's disease often die of cerebral hæmorrhage because their arteries are degenerated and their heart large and strong. The same lesion is brought about by syphilis, which causes arterial degeneration at quite an early age.

When a large artery in the brain ruptures the patient usually falls down in a state of complete unconsciousness. The face is flushed, the breathing stertorous, and the pulse slow and strong. The pupils are often unequal. Such are the characteristics of an apoplectic seizure, but, in practice the hæmorrhage most often takes place in those parts of the brain which conduct the motor impulses, and as these parts are rapidly irritated and destroyed by the gush of blood, various motor symptoms accompany those which were mentioned above. Also, as the hæmorrhage most frequently occurs in one hemisphere, it follows that the motor symptoms are usually present in one half of the body only; of course in the half opposite to the affected hemisphere on account of the decussation of the motor fibres. If the hæmorrhage be in the pons where the fibres from both hemispheres lie close to each other, the motor symptoms may be on both sides; and if it be very large it may so tear up and compress all parts of the brain that the paralysis may be widespread.

Now in a few rare cases convulsive movements occur at the onset of the apoplexy and the features may twitch and the limbs jerk to and fro. Here it is essential to note whether both sides are convulsed or one side only; or if the face, the arm or the leg be the only part which is moved. Convulsions are most commonly met with when the hæmorrhage is in the pons or in the motor centres on the surface of the brain. More commonly a movement called "conjugate deviation" of the eyes accompanies the stroke. This means that both eyes are turned strongly to one side or the other for a longer or shorter time; and as in most cases, the eyes turn to that side of the brain in which the hæmorrhage is going on, it is important to note whether they look to the right or the left. In some cases the whole head is twisted round at the beginning of the fit.

The convulsive movements are, as has been said, rare and they are transient, so that they will only come under the observation of a nurse if she happen to be present at the commencement of the fit. On the other hand, in a case in which the seizure is severe, the paralytic symptoms come on rapidly, and they persist, as a rule, even after the unconsciousness has passed off, and very often for the rest of the patient's life. The part of the motor path which is most frequently the seat of hæmorrhage is the part called the "internal capsule," and when the hæmorrhage does occur there the resulting paralysis takes the form of "typical hemiplegia" which was described in the last lecture.

Now when the patient has recovered consciousness it is easy to recognise the presence of hemiplegia. He cannot move his arm and his leg. Also the affected side of the cheek is flatter and smoother than the other, and, if asked to show his teeth, the affected side of the lip remains motionless. Lastly, if the tongue be protruded, it points to the paralysed side, being pushed over by the stronger muscles of its unparalysed half. On the other hand, while the patient is unconscious, it is difficult to detect the paralysis. If the limbs be lifted and allowed to drop back upon the bed it is found that the paralysed limb drops more limply than the sound one. But the face gives better indications of the muscular weakness, for the lower corner of the lip may droop on the affected side, while the sound corner may be a little drawn up. Also the affected cheek is flatter than the other, and is often puffed out during expiration. Inequality of the pupils also points to hæmorrhage into one hemisphere. The temperature should always be taken on both sides, for it has been found that it is usually rather higher on the paralysed side. In old cases of paralysis, however, the paralysed parts are colder than the sound ones.

During an apoplectic fit a nurse must see that the patient's breathing is not impeded by clothing or pillows, etc. She should, if possible, clear the mouth and throat every now and then of the mucus which accumulates there, and she must watch the symptoms closely. The temperature must be taken every hour, because in certain cases (chiefly when the hæmorrhage is in the pons) it rises rapidly to 106° or 107°, which usually indicates that death will soon occur. If the coma be prolonged, feeding has to be undertaken and every care must be taken that no drops get down the larynx. Some patients die after a day or two without recovering consciousness. Others, after a day or two, show some signs of consciousness but relapse again and die, in about a week, usually with a high temperature, which indicates pneumonia or suppuration round the blood clot in the brain. Others regain consciousness after a few hours or a day or two, and eventually recover but remain hemiplegic. In others, even the paralysis may pass off partially or almost completely, the hand being the part to recover least. Anything like good recovery would, however, be very unlikely in so grave a case as I have described.

If a small cerebral vessel break and the blood leak out slowly the coma may be preceded by pain in the head, giddiness, noises in the ears, flashes of light, vomiting, etc. If the leakage be very slight, consciousness may soon return. If there be no hæmorrhage but a blocking of a cerebral artery by a clot, there may be hardly any coma, and the hemiplegia may come on in the course of some minutes. If embolism occur the hemiplegia may be instantaneous but absolutely without any loss of consciousness. Hence every premonitory symptom of apoplexy must be watched, as each may give some indication of the position or the nature of the lesion. When the patient can speak, or attempt to speak (either before, during, or after the fit), his method of speech is important. Some cannot speak because they cannot use their muscles of speech, and cannot, therefore, enunciate words. Others start a sentence correctly but soon jumble up the words so that they become unintelligible. Others, again, pronounce clearly but have forgotten the words, the names of things, etc. They may, however, recognise the words when they hear them. Such patients often pronounce one word, or one short phrase, whatever they may wish to say, and, as sometimes they recognise that they are saying the wrong thing, they are much distressed. Each of these methods of speech has its own significance.

### Appearance and Nursing of Small-pox.

EXAMINATION QUESTIONS FOR NURSES.

Result of November Competition.

The question was as follows:—(1) What symptoms would cause you to suspect a case of small-pox? (2) State the difference between a slight and a severe case. (3) What are the chief difficulties to be encountered in the nursing? How would you feed the patient?

First Prize.

1. Severe headache, with pains in the back, and rise of temperature. A rash resembling measles, appearing usually first on the face and arms; the spots soon became hard and feel to the touch like small shot under the skin. In four or five days the spots begin to maturate and thus form pustules, which discharge and then gradually dry up, forming a crust.

2. In a slight case the pustules are isolated, it is then termed a discrete case. In a severe case the patient is covered with pustules which run one into another, it is then termed confluent. There is very high temperature, and often violent delirium. In a case terminating fatally, death occurs from exhaustion, or from one of the complications of small-pox; the chief of which are pneumonia and laryngitis, the latter caused by the pustules forming on the throat.

3. It is sometimes very difficult to count the pulse of a small-pox patient on account of the pustules. The eyes require careful and frequent bathing, as the lids swell greatly and are very painful. The skin cannot be washed until the pustules begin to dry. The diet must be nourishing and plentiful, milk, beef-tea, strong broths. Brandy is often ordered in severe cases. Rac.

Second Prize.

A temperature over 100°, with intense backache, sore throat, vomiting, headache, with a rigor, particularly if an eruption appeared after three days on the face, neck, and wrists, and the spots were hard.

A slight case is one in which the pustules are scattered singly over the body. There is not so much sore throat, no epistaxis, and no delirium worth worrying over. Food is taken well, and patient has no abscess.

A severe case is either

1. Confluent (pustules run one into another forming large blisters on feet, hands and other parts of the body), very severe.

2. Hæmorrhagic (tiny blue spots appear among the pustules).

3. Malignant hæmorrhagic (epistaxis and rectal hæmorrhage, pustules do not "come out" properly). General blue appearance of the skin. Usually fatal.

In the mild form there are not many difficulties. Sometimes patients get a fixed belief that they are not going to recover, when every effort should be made to reassure them, and care taken that a proper quantity of nourishment is taken.

In severe cases delirium often occurs, and difficulty is experienced in keeping patients warm and in bed. They should not be left alone, as they may escape and wander some distance, ultimately dying of exposure. It is often difficult to make patients swallow, owing to their sore throats. Drinks should then be given hot, and (if the condition of patient admits) gargles should be used, or the throat fomented with hot flannels.

Difficulty is experienced in keeping patients clean, as when the pustules break, the offensive discharge invades eyes, nose and ears. Sponging with tepid water (in which a little disinfectant has been placed) is the rule then.

The eyes should be well bathed with warm boracic lotion.

If patients try to scratch their faces (through the intense itching) the hands should be well wrapped up in oiled lint, and bandaged, while the face should be painted with lanolin, olive or carbolic oil, which will moisten the scabs, and mitigate the itching.

Lung, throat, kidney, and (*very* often) eye troubles are complications of small-pox, and sometimes blindness occurs through pocks coming on the pupil of the eye.

If there is much difficulty in swallowing small feeds (℥ iv.) every two hours, of milk, beef-tea, chicken broth, egg and milk (and stimulants when great weakness), at first. If liked, and able to swallow well, bread and milk, soups, bread and butter, milk puddings, fish, chicken, and any light invalid diet may be given, after the first few days. Owing to the presence in the throat, mouth and on the tongue, of the pocks, swallowing is often very painful, so that whatever food is taken best and most easily should be given. If very high pyrexia milk should be given alone, with barley water, thin arrowroot (made of milk), thin gruel to vary the monotony. When temperature is down, ordinary diet, unless great weakness. "Austral."

Honourable Mention.

This is gained by three nurses—"Nurse Manningham," "M. M. M. B." (pseudonym impossible to decipher), and "Nurse Louie." Nurse Manningham would have gained a prize if she had been a little clearer as to the first appearance of the rash. "Nurse Louie's" paper is excellent in substance, but so carelessly written and badly expressed that it could not be printed without correction, which is against rules.

The Two Best Papers.

The two prize winners this month are very naturally those who have had personal experience in nursing small-pox. "Nurse Rac" takes the first because she explains that frequently the rash on first appearing resembles to a certain degree that of measles. It is very important to know this. Ignorance of this likeness may cause a district nurse, for instance, to allow a child to associate with other members of its family, whereas had she any reason to suspect small-pox, it would be her manifest duty to take some steps towards isolating the case till medical advice could be obtained. Sometimes this preliminary rash resembles that of scarlatina. Taylor, in his "Practice of Medicine," says:—"Early eruptions.—These are either *erythematous* or *hæmorrhagic*. Of the erythematous rashes, some cover the whole body and face, and either closely resemble scarlatina, or are more like measles; in other cases the rash is partial in its distribution, and has been especially noted on the external surfaces of the arms and legs. Of the hæmorrhagic rashes the most characteristic is one which occupies the lower half of the abdomen, from the umbilicus downwards, covers the groins and extends on to the thighs in a triangular form . . . it also frequently appears in the axillæ and on the adjacent parts of the arms and trunk, and extends thence along the flanks to the lower patches. It consists of small hæmorrhagic spots or petechiæ, which, on fading, leave brown or yellowish-brown stains for a time. These initial rashes commonly appear on the second day and last for about two days, co-existing, perhaps, with the early stage of the pustular eruption, but disappearing before its full development."

"Austral's" answer is excellent. It is a pity that she failed to note this early eruption.

No Correspondence.

The questions are put in the most absolutely simple language, so that they may be suitable to all capacities. No further explanation can be given. The simplicity of the questions we are now considering is surely such as to need no interpreter!

Question for December.

1. How would you proceed to undress a man suffering from a simple fracture of the right forearm? 2. What difference would you make if the fracture was compound? 3. How would you proceed to undress a man with a simple fracture of the left femur? What measures should you take if the fracture was compound?

N.B.—Do not repeat the questions at the head of the papers, and write simply, not in complicated paragraphs.

The Examiner.

### Our Christmas Distribution of Clothing.

The contributors to our Clothing Distribution will be glad to know that the number of garments received this year greatly exceeds that of last, the total being upwards of 250. It is a satisfactory feature that the quality of the articles shows a distinct improvement. We specially desire to commend the natural wool hospital jacket, which buttons from the shoulder to the wrist, so as to save fatigue to the patient in putting it on. There are also some charmingly soft woollen vests of the ordinary shape, and a large quantity of knitted socks, beautifully made. Most acceptable to a patient leaving hospital will be the warm woollen slippers which one of the parcels contained. The shamrock sent by "M. R.," another contributor, was greatly appreciated by the staff.

The following is the list of contributions not hitherto acknowledged:—Miss Annie Burt, Lelant, Cornwall; Miss Henslowe, Preston Vicarage, Weymouth; Miss Macdonald, 24 Neville Street, Onslow Gardens, S.W.; M. A. Stout, Mill Road Infirmary, Liverpool; Nurse Richardson, Cowbridge-on-Tyne; Clara Cooper, Chevington, Bury St. Edmunds; Margaret (aged 10); Sister Atten, 51 West Hill, Sydenham; Miss Hitchcock, The Beeches, King's Lynn; G. K. L., Prestbury, Gloucestershire (who encloses some scarves made by a patient nearly blind); M. R., Ryde; Nurse Ellen Clarke, 36 Porchester Square; Miss Pritchard; and the Editors of The Hospital, £1.

The garments have been sent to the following hospitals:—Tottenham Hospital, University College Hospital, Evelina Hospital, Poplar Hospital for Accidents, North London Hospital for Consumption, Grosvenor Hospital for Women and Children, Royal Free Hospital, Middlesex Hospital, the London Hospital (children's wards), Metropolitan Hospital, Kingsland Road; Westminster Hospital, North-Eastern Hospital, Hackney.

### The Nurses at St. Olave's Infirmary, Rotherhithe.

By a Correspondent.

Certain damaging statements having appeared in one of the London daily papers about the condition of the nurses at St. Olave's Infirmary, Rotherhithe, I have made careful inquiries on the various points raised.

The article was headed "Overworked Nurses: Poor Accommodation and Long Hours," and it was stated that complaints had reached the paper to the effect that the nurses at St. Olave's Infirmary were so much overworked that many of them had thrown up their appointments.

The first charge was that some of the nurses had alleged that the infirmary was understaffed, and the article further stated that Miss Mary Simmons, one of the Guardians, had admitted this, and that she placed the blame upon the Local Government Board. She was represented as saying, "We have asked several times for permission to increase the staff at St. Olave's, but the Local Government Board says that we are staffed up to our needs, and, therefore, while recognising our wants, we are helpless to move further in the matter."

On putting this matter before Miss Simmons lately, I was informed that no blame whatever attached to the Local Government Board, who had, as a matter of fact, recently sanctioned the engagement of four charge nurses.

Further, Miss Simmons said that one nurse to 11 patients, the proportion at St. Olave's, could not possibly be called "understaffing." From the Clerk of the Board (Mr. Pitts Fenton), it was ascertained that the exact number of nurses, counting the matron, two assistant superintendents, and one night superintendent, was 61, the number of patients being 640. He said he had never known the Local Government Board refuse to sanction additional probationers when asked to do so, and he had been in his present office ten years; moreover, another application had just been sent up.

"Ill-housed" was the next grievance; and on this matter both Miss Simmons and Mr. Pitts Fenton were emphatic. The Nurses' Home consists of a two-storeyed building apart from the main block; in this each nurse has her separate room. There is a sitting-room on each floor, and 36 nurses have accommodation in this building. Four charge nurses, who are on night duty, have to sleep in lodgings outside; the Guardians allow them 10s. each per week over and above their pay for this little discomfort. Of the rest the matron, two assistant matrons, and night superintendent have their own rooms, and about seventeen probationers sleep in a cubicled dormitory or "associated room" in the main building. The Guardians, I was informed, have in hand a scheme, which comes up every six months for consideration, for building a new Nurses' Home, so that all may have the privilege of a separate room. It will, however, be a very heavy expense of some thousands of pounds; and so much building has been absolutely necessary of late that it is not felt right as yet to impose this additional burden upon the ratepayers, though as soon as possible it will be done. Miss Simmons is of opinion that hardly a Union in the Metropolis would be found better off than St. Olave's in this respect.

Next, the nurses who complained said they were "anything but well fed"; and the article added, "As to the alleged insufficiency of food, Miss Simmons said that any nurse had the right to complain to the Committee." Not only so, but both Miss Simmons and the Clerk asserted that there had been no complaint about the food for years. Quite recently the kitchen has been improved, and this department placed in the hands of the matron; and a glance down the "rations scale" is sufficient to show that there is no stint of food, the only difference between the allowances for the nurses and those for the matron and medical staff being that the latter have poultry, and the former only meat, fish, and rabbits. The other items of food are identical. One pound a day, of all the main articles of diet, is allowed, and other foods are in proportion.

Finally, it was said that 20 nurses had resigned in consequence of bad conditions. The actual number appears to be eight; and the cause of leaving has in neither case had any connection whatever with the conditions of living at the infirmary. Six nurses were dismissed some time ago, Miss Simmons told me, because it was absolutely impossible to keep them as a matter of discipline; two charge nurses resigned because they had obtained appointments as private or district nurses; and any other changes are accounted for by the fact that nurses, having finished their training, have preferred taking up work elsewhere; but even of these, there has been no number recently. "The statement that twenty have left," said Mr. Pitts Fenton, "is not true."

As to the long hours, Mr. Pitts Fenton remarked that it would require a very bold Board of Guardians to introduce the system of "an eight hours' shift," that is, three nurses to the twenty-four hours. Miss Simmonds admitted to the representative of the paper containing the statements in question that her personal opinion upon the matter was that all nurses worked too long at a stretch; but on this point there does not seem to be the slightest reason to single out St. Olave's Infirmary, rather than any other, in the Poor Law Union.

### The Pension Fund Souvenir.

The proprietors of The Hospital have been much gratified at receiving so many letters from nurses expressing approval of both the album and the plate. They think one or two quotations may be interesting to those who have not yet seen the souvenir.

Nurse A. S. H. writes: "Album and plate duty received both beautifully got up."

Policy No. 2,071: "I wish to acknowledge with many thanks the receipt of the beautiful souvenir, with which I am much pleased. I should think every nurse who was present at Marlborough House would like to possess one of these extremely interesting and handsome souvenirs."

Policy 7,381: "Many thanks for plate, and am very pleased and proud to have it, and sincerely thank you for the trouble taken on our behalf, as all the nurses are, I feel sure."

The Press endorse the praise given by the nurses. The *Times* calls the souvenir "a handsome volume." The *Ladies' Field* says: "A very charming souvenir . . . the letterpress is very interestingly written, whilst excellent collotype plates are mounted for framing." The *Queen* writes: "This very beautiful souvenir. . . . It is a very faithful record of days which will linger long in the memory of all present. The portraits are of special beauty."

Great popularity has often some drawback, and this is so in the case of the plate which portrays the ceremony of the presentation of certificates. The demand has largely exceeded expectation, consequently the proprietors crave the indulgence of those nurses who have not yet received the ordered picture. Very careful printing is necessary, and it is impossible to hurry the production. Nurses must therefore please understand that their orders have not been overlooked should the picture not reach them by return of post, There will be no delay in the matter of the albums, as these were prepared in larger numbers. The proprietors take this opportunity to reiterate that the purchase of the works is open to all, until the edition is exhausted.

### Opening of the Victoria Nurses' Home, Northampton.

On Thursday, last week, the Princess Christian visited Northampton, and opened the Victoria Nurses' Home, Northampton's permanent memorial of the diamond jubilee of Her late Majesty Queen Victoria. Princess Christian, who was accompanied by her daughter, Princess Victoria of Schleswig-Holstein, after receiving a loyal address from the Mayor and Corporation at the Town Hall, drove in procession to the Nurses' Home.

A heavy snowstorm, followed by equally heavy rain, greatly marred the decorative signs of welcome, and prevented many people from being present. At the home, however, the arrangements were so well planned even the inclement weather did not spoil the programme. The space in front of the building was cleverly utilised to form a large enclosed stage with galleries on either side. The decorations of red, white, and blue were most effective, lighted up by festoons of electric lights. On the arrival of the Princess, accompanied by a large party from Althorp Park, the seat of Earl Spencer, she was conducted to a daïs, where was placed a gilt chair specially made for Queen Victoria at the opening of the Great Exhibition in Hyde Park in 1851.

After the presentation of a bouquet to her Royal Highness, the Bishop of Peterborough commenced the ceremony by reading a special prayer. Addresses were then presented from the Memorial Committee and the Nursing Institute respectively, to each of which the Princess replied, expressing her approval of such a suitable memorial "to my beloved mother, Queen Victoria," and alluding to her own personal interest in "nurses and their work for the past 20 years." The presentation of purses by some thirty prettily-dressed children, containing upwards of £170 towards the funds of the Home, was the next part of the function.

The Princess was then presented with a key of the Home, and proceeded to unlock the central door and declare the building open. As her Royal Highness entered she was received by the lady superintendent, Miss Lunn, the assistant superintendent, Miss Ross, the four Jubilee nurses, and the private staff, forming a picturesque group in their pretty uniforms. The Royal party, conducted by the lady superintendent, at once proceeded to inspect the Home, which is a charming building. It is three storeys high, faced with stone, and a roof of red tiles. The secretary's office, superintendent's room, and the nurses' dining and sitting rooms are on the ground floor. These were not seen in their ordinary conditions, as they were necessarily rearranged to admit of the large company to tea after the departure of the Princess. They are appropriately though simply furnished, and are heated by hot water in addition to tiled fireplaces. They were specially ornamented with plants and flowers on Thursday, and the general effect was very pleasing.

On the first floor are the superintendent's bedroom, three rooms for private patients, a well-stocked linen cupboard, two bathrooms, and housemaid's sink, all fitted and furnished in the same simple yet most comfortable and efficient manner. On the second floor are seven bedrooms and a bathroom; the bedrooms are very pretty, and every nurse has her own separate one. In the basement are the kitchen and scullery, a box-room, pantry, larder, storeroom, and coalcellar, and a large "district room," fitted with sinks, a hand-basin with ample supplies of hot and cold water, cupboards for the nurses' cloaks, bags, etc., and a most complete set of cupboards for all the appliances for district work. These are well stocked in every particular, both for the nurses' needs and for lending to the patients. With so commodious and well appointed a home it is not surprising that the visitors expressed unqualified approbation of all they saw.

Princess Christian consented to become the patroness of the institution, and Princess Victoria laughingly said she would not object to occupy a patient's room herself. Princess Christian also expressed her pleasure at finding Miss Lunn, formerly one of her nurses attached to the Princess Christian's Home at Windsor, at the head of such a splendid institution.

After signing their names in the Visitors' Book the Royal party left, and the spectators of the ceremony were entertained to tea and inspected the Home. The most favourable comments were expressed with regard to it on every side, and the general feeling was one of entire satisfaction. Amongst those present were Miss Peter, superintendent-general of the Queen Victoria's Jubilee Institute, and Miss Amy Hughes, superintendent of the work connected with the County Nursing Association. The committee, Miss Lunn, and her staff are to be congratulated on the successful way in which all was conducted. The preparations entailed much hard work on all concerned, but the result left nothing to be desired.

### Everybody's Opinion.

### WHY ARE NIGHT NURSES NEGLECTED?

"N. G." writes: I am glad to see that all matrons do not agree with "Yorkshire" when she says that nurses are degenerating, because they ask for a little variety in food, and the common comforts of everyday life. I know that in many hospitals the food for the nurses is excellent, as well as all the arrangements for their comfort, but in some there is still much to be desired. During the last few months of my time spent in hospital, I often went on duty on cold winter mornings with nearly no breakfast, because there was not enough to go round. In the same institution I have seen a nurse who had breakfasted at 6.30 A.M., come down to second dinner at 2 P.M., and because there was no meat left (she being a little late), she dined on some potatoes, and they were burnt, and the pudding that followed was such that no one could eat it. Was it to be wondered at that complaints were frequent, also that the nurses were constantly knocked up? There is a standing joke against me to this day in my own home circle that my sister wrote to me saying that the garden at home was lovely, and asking if I would not like some flowers. My reply arrived in due course written on a post-card, "Thank you so; flowers are pleasant to the eye, but not good for food; and how to get enough to eat is the only thing I can think of at present." Let this speak for itself.

---

### THE "ENGAGED" PROBATIONER.

"An 'Unengaged' Nurse" writes: A medical man recently, in giving the introductory lecture of an elementary course to the probationers of a provincial training school, surprised his audience by the following remark:—"A young woman who is engaged to be married should not be accepted as a probationer in a training school. The first reason is, that she crowds out those who intend to make nursing their profession. Another important reason is that an engagement gives her a strong interest outside the hospital wards which is likely to absorb the best of her faculties." It is to be feared that the remainder of the lecture was not listened to with that rapt attention which it is the bounden duty of the new probationer to bestow on the dicta of the doctor appointed to initiate the budding nurse in the elements of surgical anatomy. Several among the newly-admitted pros. were secretly conscious of a jewelled ring erstwhile on the third finger of their left hands, and now consigned to a cotton-wool oblivion in their trunks. All of them were at once "convicted" of their sin in not mentioning the interesting fact to matron when she "passed" them as fit candidates for the nursing ranks. The sentiment set me thinking. Should an engaged girl forego the valuable training for wife and motherhood afforded by a three years' course in a hospital for the sake of an abstract, ethical fear of crowding out a woman who intends to make nursing her profession? Does not the benefit which accrues to the community and family through the maternal head of a household being a skilful nurse outweigh a mere trades-union consideration? I confess to a sense of puzzledom on this subject. There are so many points of view. And is there any really valid reason why a girl engaged to a man who is unable to marry her for some years should not fill up the space of waiting by a course of training which would admirably fit her for that state of matrimony to which she will eventually be called? This is a knotty and interesting problem. Three matrons of leading training schools to whom I appealed were emphatic and unanimous. "We should not think of admitting a probationer if we knew her at the date of entrance to the hospital to be engaged." This appears to be the matron's professional point of view. The head of a well-known art school in England promptly asks each of his feminine pupils who become engaged to cease attendance at his school. "I train artists—not wives" is his famous epigram. Is this also to be the sentence of the training school? "We turn out professional nurses—not wives and mothers." "It seems such waste," said an experienced sister-in-charge, "after taking years of trouble to teach a girl to be a good nurse, to find her marrying straight from the training school." Knowing what a household treasure to husband, children, servants, and the community generally, a trained nurse usually proves after marriage, I wondered whether it is such "waste" to develop a mere married woman! "Engaged girls ought to enter as paying probationers," says another hospital matron. But the engaged girl may be poor, notwithstanding that she is imbued with a desire to fit herself for the care of a future household and family. And do not her services during the training period reimburse the hospital for the outlay in time, trouble, and keep, involved in the production of a certificated nurse? Further, is there any implied promise on the part of a nurse that she is necessarily intending to follow her vocation on purely professional lines? Is an engaged girl in any sense put upon her honour to "declare her intentions" as to marriage at the time she enters a training school. It would be extremely interesting to learn the views of matrons, sisters, and nurses on the many issues that have been opened up by the original remark quoted in the beginning of this letter as being made by a medical lecturer to his class of probationers.

### Appointments.

Birmingham Infirmary.—Miss Hilda Solley has been appointed night superintendent. She was trained at Birmingham Infirmary, holds the L.O.S. certificate, and has been for three years sister of a female surgical ward, besides doing temporary duty as night superintendent and house sister.

Brentford Union Infirmary, Isleworth.—Miss Louisa Richford has been appointed assistant matron. She was trained at St. Marylebone Infirmary for three years. She has since been ward sister at the Brentford Union Infirmary for 18 months; ward sister, and then home and theatre sister, at the Bradford Union Hospital for the last two years.

Chester General Infirmary.—Miss Edith Addis has been appointed lady superintendent. She was trained at the Nightingale School, St. Thomas's Hospital. She has since been in charge of the male and female blocks at Bolton Union Infirmary; night superintendent at Radcliffe Infirmary, Oxford; and assistant superintendent at Leeds General Infirmary.

Hebburn-on-Tyne Infectious Diseases Hospital.—Miss Jeannie Sutcliffe has been appointed matron. She was trained at Bradford Royal Infirmary and Beckett Street Fever Hospital, Leeds. She has since done private nursing and been matron at Mirfield and Liversedge Fever Hospital.

Oxford Union Infirmary.—Miss Agnes Kinnear has been appointed assistant nurse. She was trained by the Meath Workhouse Nursing Association at the Royal Hospital, Sheffield.

St. Leonard's Infirmary, Shoreditch.—Miss Selina A. Clifton has been appointed ward sister. She was trained at the Metropolitan Hospital, London.

The General Hospital, Birmingham.—Miss Emily E. Roberts has been appointed staff nurse. She was trained for three years at the Brentford Union Infirmary, Isleworth.

Walsall and District Hospital.—Miss G. L. Guest has been appointed charge nurse. She was trained at the Metropolitan Hospital, London, for three years, and has since been staff nurse and holiday sister in the same institution.

### Presentations.

Sanatorium, Halfkey, Malvern.—Miss A. E. Holtom, on her retirement from the post of matron, was presented by the Malvern Urban District Council with a handsome mahogany grandfather's clock, also an illuminated address; by the officials with a chiffonier, and by the nurses with a very pretty tea-service. The address was neatly framed, and contained a graceful allusion to Miss Holtom's approaching marriage.

The Lyndhurst District Nurse.—The committee of the Lyndhurst Nursing Association have presented Miss Clutterbuck with a cheque for £10, as a token of their appreciation of her devotion to her work in the district during the past seven years. Miss Clutterbuck was trained at the Birmingham Infirmary, and holds the L.O.S. certificate. She began her work at Lyndhurst in December, 1894.

### Where to Go.

Grand dramatic and musical performance at the Wellington Hall, Eyre Arms, St. John's Wood, in aid of St. Mary's Hospital, Jan. 20th, 1902.

### Wants and Workers.

"The Hospital."—Would any nurse like The Hospital posted Monday after publication? If so, please send name and address to Lillie, 6 Woodside Terrace, Douglas, Isle of Man.

### Echoes from the Outside World.

### AN OPEN LETTER TO A HOSPITAL NURSE.

One is so accustomed to look upon the clothing of men as of too prosaic a character to be of any interest, except to the wearers, that one learns with a little shock of surprise that it is the King himself who will be the central figure of the Coronation ceremonies. He is, it is said, to wear a cloth of gold under-jacket, embroidered with palm branches, and the rose, the shamrock, and the thistle. But it is upon His Majesty's cope that the greatest amount of gorgeous embroidery will appear. The three floral emblems of England, Ireland, and Scotland will be introduced, woven in gold thread, together with the royal crown of the Cross of St. George, silver eagles, and fleur-de-lys. The crown jewels will of course be displayed in their full magnificence. Meanwhile, the route of the procession, to take place on the day before or after the 26th, is reported to be settled, but it is not official, and those who regret that the streets specified do not include the only houses where they have friends who might invite them have still room for hope, and those who are now congratulating themselves may have cause for despair. At present Regent Street has not been placed on the list of the proposed route, and stupendous efforts are being made to secure its inclusion. The Royal Proclamation as to the date of the Coronation, the new coinage, the arms of the Prince of Wales, and the meeting of Parliament, was read by the Common Crier from the steps of the Royal Exchange on Thursday, and notwithstanding the pouring rain a considerable crowd assembled. There are to be new pennies, half-pennies, and farthings, also £5 pieces, £2 pieces, sovereigns, and half-sovereigns. The new issue of silver money is not yet ready because it is not yet required.

---

The news from South Africa which was published on Monday made a bright beginning to a week which, at home, will be full of busy preparations for a festival of Peace. General Bruce Hamilton and his men, for the second time in seven days, covered 50 miles in 24 hours, and surprised the enemy near Ermelo so completely that they took many prisoners and cattle, and also recaptured one of Benson's guns, which was in such good order that it was used upon the retreating Boers. Commandant Badenhorst was taken prisoner. The Government evidently think very highly of General Bruce Hamilton's feat, for the Secretary of State for War has despatched the following telegram to Lord Kitchener: "Please convey the hearty congratulations of his Majesty's Government to General Bruce Hamilton on his brilliant achievements." The progress towards a satisfactory conclusion of the war is abundantly shown by the fact that owing to the erection of blockhouses, a practically clear country for 100 miles radius round Johannesburg has been secured. Consequently, early in the new year some of the mines will be in working order, and the Stock Exchange will be open once more.

---

Lord Rosebery must have felt very tired when he got back to London on Monday night. His journey to Chesterfield and back was the least wearisome part of his expedition. But to speak for two hours in an immense hall, and know that a large number of his hearers could not hear, while those who could hear were eager to criticise, is a tremendous strain even on a man in the prime of life and the fulness of his powers. Lord Rosebery seems, however, to have got over his task without any ill effects, and to have at least succeeded in convincing everyone that his proper place is not the pleasant seclusion of the Durdans, but the stormy arena of politics. Of course, he disappointed the persons who imagined that he was going to make some sensational announcement, and he said much that was highly controversial. Nor does it seem in the least likely that his dream of the formation of a new central party will be speedily realised, if ever. But he treated his topics with so much vivacity, so much earnestness, and so much grace of language that whatever retorts he may have exposed himself to, we must all be glad to reckon him as a real force in the country, instead of regarding him in political matters as an extinct volcano.

---

You will learn with pleasure that at the meeting at Guy's Hospital of presidents of the League of Mercy on Monday the treasurer was able to congratulate the workers of the League on the amount obtained during the present year. The first year the organisation was able to contribute £1,000 to the Prince of Wales's Hospital Fund, the second year £6,000, and this year it hands over £7,000 as the result of the labours of its members. And this is the more satisfactory because the subscriptions did not come from London alone, but from many districts stretching far into the country, thus telling a tale of wide-spread and increasing interest. Another notable point referred to was that there have been few changes in the *personnel* of the League, which shows that those who joined it at the outset have, with very few exceptions, continued to work and to increase the amounts in their districts.

---

I should like to hear what the opponents of the policy of muzzling dogs as a prevention of rabies have to say about the latest order on the subject? The Board of Agriculture have issued a memorandum stating that there is now no necessity to enforce muzzling throughout the United Kingdom, and dogs arriving from Ireland need no longer be detained in quarantine, because "there appears to be good reason to believe that rabies has now ceased to exist in any part of the United Kingdom." I fancy that even those hot-headed persons who were once desirous of tearing Mr. Walter Long limb from limb, must admit that the discomfort their pets suffered under the order, was worth the present immunity from a dreadful disease. But as rabies is still very prevalent on the Continent of Europe, and in most other parts of the world, dogs landed from abroad after March 15th will have to be detained and isolated at the expense of their owner under the control of a veterinary surgeon for six months subsequent to their arrival on these shores. Thus should the recurrence of a fresh outbreak be rendered unlikely, if not impossible.

---

Of course electricity has taken an important part in the gifts for the season, and quite one of the most delightful is a small clock for a bedroom. It has within its stand a small battery to which a coil of wire is attached. The clock stands on the mantelpiece or elsewhere, the end of the coil lies beside the pillow of the sleeper. When she awakes, and is anxious to know the time she presses a button, and the face of the clock is at once illuminated without any further trouble, so that the constant striking of matches or the lying awake to listen for the chiming hours is avoided. The charm of the design is that it is equally useful as a masculine or a feminine gift, and to many nurses it would be most useful. There is also a walking stick on show now possessing a tiny illuminating power in its top, which might be very handy at times, but should be given with care lest the male recipient should read in it an insinuation that he sometimes finds it difficult to insert the latchkey into the door when returning from a dance or a dinner. Amongst the unelectrical presents for musical friends, photograph frames are to be had with a miniature page of a song enamelled on the front, the face of the photograph peeping out of the centre of a line. The titles of the songs are varied as much as possible, so as to be applicable to different people, "For Auld Lang Syne," "My Heart's Treasure," etc.

### A Book and its Story.

### FAMOUS WOMEN.*

"Little Journeys to the Homes of Famous Women" is the third volume in a series of short biographies of eminent persons, written by an American, and containing twelve sketches of the lives of well-known women of the eighteenth and nineteenth centuries. These are for the greater part Englishwomen, distinguished in the world of art, letters, philanthropy, and politics. The latter term applies particularly to two women who, though gifted to an unusual degree, the one with personal beauty and charm, the other with intellectual powers in addition, were not Englishwomen, viz., the Empress Josephine and Madame de Staël. The sketches are interesting as being the result of impressions made in the immediate vicinity of places identified with the subjects of them, either where they were born or had lived during the more important part of their lives. Mr. Hubbard writes with caustic brevity, and if the reader is reminded occasionally that American English is not what is known as "Queen's" English, and that he applies an interpretation other than that in use among us, we must remember that these little journeys were made to shrines distant from his own country, and that they were written primarily for his countrymen at home who will see nothing to condemn, but much to commend, both in matter and style.

Each journey is prefaced by a portrait of the famous woman who is treated therein, and at the head of the list stands Elizabeth Barrett Browning, followed by Madame Guyon, Harriett Martineau, Charlotte Brontë, Christina Rossetti, Rosa Bonheur, Madame de Staël, Elizabeth Fry, Mary Lamb, Jane Austen, the Empress Josephine, and Mary Wollstonecraft Shelley. The biographical sketches do not dwell on the works with which many of the above names are indelibly associated; they treat simply of everyday incidents common to the lives of most men and women; only incidents in commonplace lives become episodes of far-reaching importance, when the actors are men and women of genius. To the hundreds of readers to whom Browning as a poet appeals, there are thousands to whom he appeals not at all, but everyone has ears for the touching story of his love for Elizabeth Barrett, and any biographical notice would be incomplete without reference to it. Here it seems to stand out, as it undoubtedly was, as the happy turning point in the life of Elizabeth Barrett; and the blending of two kindred souls was more perfect as a union than that of any other poet's of the century. Those lovely sonnets from the Portuguese, the most exquisite love-poems, were, as everyone knows, the work of Elizabeth Barrett, inspired by her devotion to Robert Browning, "under whose phlegmatic outside there was a spiritual nature so sensitive and tender that it responded to all the finer thrills that play across the souls of men. . . . The love between Elizabeth Barrett and Robert Browning is an instance of the Divine passion; they had passed well beyond their first youth; there was a joining of intellect and soul which approaches the ideal. . . . Each one at once felt a heart rest in the other. Each had at last found the other self." The ideal reduced to the real has to face every-day facts, and Robert Browning had to confront a stern and tangible fact in the person of his future father-in-law. "But Browning had—in spite of being a poet and in love—an even pulse, a calm eye, and a temper that was imperturbable. His will was quite as strong as Mr. Barrett's." So one day without the consent of her obdurate father, Elizabeth met her lover—the author describes it as "on the street corner" —and in a very prosaic way they took a cab, drove to Marylebone Church, and were married, the bride returning home alone. It was a week before they met again, when the gentle Elizabeth stole out leading her dog Flush, and she and her husband crossed to Calais that day. They wrote the next day asking forgiveness, but no forgiveness was forthcoming from the irate Mr. Barrett.

Of Rosa Bonheur, the author has some interesting things to say. She was born early in the century at Bordeaux, and her father never quite forgave her for not being a boy. "A son was expected—a first born—an heir. There was not anything to be heir to, except genius, but there was plenty of that, and the heir was to bear the name of the father—Raymond Bonheur. The days were fulfilled—the child was born. The heir was a girl." Raymond Bonheur was a struggling artist, and he never prospered until he moved to Paris and copied pictures in the Louvre. These sold, and he was able to make a home for his four motherless children. Rosa was apprenticed at 12 years of age to dressmaking. But she rebelled at the uncongenial calling and begged her father to instruct her. From childhood she had drawn, as children will, but always animals. Now she would learn systematically. She accompanied her father to the Louvre, and was soon copying pictures. In those days no women artists worked, and "people laughed at the little girl with a yellow braid of hair mixing paints and helping her father.' "Let's cut off the braid and I'll wear boy's clothes and be a boy," said funny little Rosalie. The next day Raymond Bonheur had a close-cropped boy in loose trousers and a blue blouse to help him. "Rosa saw that her father's efforts to please others had not been always successful. He had tried many forms. She would stick to one—she would paint animals and nothing else. All the honours of the Salon were heaped on her after the painting of her great picture 'The Horse Fair.' She was then 28. By special decision her work henceforth was declared exempt from examination by the jury of admission. Rosa Bonheur, five feet four, weighing 120 pounds, was bigger than the Academy." Mr. Hubbard visited her home at By, near Fontainebleau, and at the time of his visit, "found her at 74, taking a woman's tender interest in all sweet and gentle things, and with an imagination, in its strength and boldness, peculiarly masculine . . . beloved by all who knew her." Jane Austen, who lived a hundred years ago, the daughter, like Charlotte Brontë, of a country clergyman, an authoress whose books during her lifetime were all published anonymously, has always had admirers. Southey, Coleridge, Guizot, Macaulay, hailed her as "a marvel of insight," and her books, like those of Charlotte Brontë, being perfectly simple and consisting of human records, illuminated with fine insight into the character of real men and women who lived, and loved, and hated, as they will to the end of time even, at this distance have still power to arrest and delight. "To weave your spell out of commonplace events, and brew love potions from everyday materials is high art." "Jane Austen was a commonplace person," Mr. Hubbard considers, and "Kipling himself is quite a commonplace person also. He is neither handsome nor magnetic. He is plain and manly, and would fit in anywhere." "The Little Journeys" will make an admirable gift book, at this season, for those who may like to read of some "commonplace persons" who achieved very uncommonplace fame, and of others who were gifted and brilliant in an extraordinary degree.

* "Little Journeys to the Homes of Famous Women." By Elbert Hubbard. (Published in London and New York by Putnam and Sons. Illustrated. 1 vol. 5s.)

### For Reading to the Sick.

CHRISTMASTIDE.

Deep was the midnight silence in the fields
Of Bethlehem; hushed the folds; save that at times
Was heard the lamb's faint bleat; the shepherds, stretched
On the green sward, survey'd the starry vault.
When, hovering floats
Upon the folded flocks a heavenly radiance,
From whence was utter'd loud, yet sweet, a voice:
"Fear not, I bring you tidings of great joy;
For unto you is born this day a Saviour;"
And with the Angel, lo! upon the cloud,
A multitude of seraphim enthroned
Sang praises, saying, "Glory to the Lord
On high: on earth be peace, goodwill to men."

*J. Grahame.* 1765.

On this day the Church's watchword is "Rejoice." Her cries of sorrow give place to Alleluias. She puts on the "garment of praise," and anoints herself with the "oil of gladness," that she may join her voice with that of Angels and welcome her "Saviour which is Christ the Lord."

In heaven there is great joy. All the Angels adore Him as a Child, whom as God they had worshipped.

And there is joy in earth. For the Deliverer is come. He comes to save mankind, to redeem the world.—*From Devotional Helps.*

We all know it is the Home Festival. Now families meet, and old friends return. Now a simple, quiet human gladness comes in some way to every heart. It is the Festival of the Children, and this sad world would indeed be sad and dark were it not for the songs of the birds, and the colours of the flowers, and the fair faces and cheery voices of the children. And so it is that none of us is

"So dark in memory or sad at heart,
At such a distance from his youth in hope,"

as to forget on Christmas Day the joy of the children, and to forget that that joy is due to Christ.

Very mysterious, very moving it is, when we feel that the deepest and the simplest things in life lie side by side.

Now, so it is at Christmastide. Nothing so simple, nothing so grand, as the Christ, at once our King and Child! Nothing so high in unmeasured heights of dim elevation, and so near the simple tenderness of our own fireside!

*Knox Little.*

At the break of Christmas Day,
Through the frosty starlight ringing,
Faint and sweet and far away,
Comes the sound of children, singing,
Chanting, singing,
"Cease to mourn,
For Christ is born,
Peace and joy to all men bringing!"
"Merry Christmas!" hear them say,
As the East is growing lighter;
"May the joy of Christmas Day
Make your whole year gladder, brighter!"
Join their singing,
"To each home
Our Christ has come,
All Love's treasures with Him bringing!"

*Margaret Deland.*

### Notes and Queries.

**The Editor is always willing to answer in this column, without any fee, all reasonable questions, as soon as possible.**
**But the following rules must be carefully observed:—**
**1. Every communication must be accompanied by the name and address of the writer.**
**2. The question must always bear upon nursing, directly or indirectly.**
**If an answer is required by letter a fee of half-a-crown must be enclosed with the note containing the inquiry.**

*Hospital for Consumption.*

(115) Can you give me any information as to the best method of getting a boy into a hospital for consumption?—*A Nurse.*

Make application to any of the hospitals for consumption and fill up the forms sent, complying with the directions given.

*Boy with Diseased Bone.*

(116) Can you tell me of any convalescent home in South of England where a boy suffering as above would be received?—*W. W.*

You will find a list of several suitable institutions in "Burdett's Hospitals and Charities."

*Norwich.*

(117) In reference to the answer to *M. A. B.* last week, we have ascertained that the Norwich Nurses' Co-operation, 36 Bracondale, Norwich, is managed by a committee of ladies, and worked on co-operative lines.

*Maternity Nurse and Trained Midwife.*

(118) If Nurse Jones still wishes for a private reply to her question she must observe the rules as given above.

*Lectures.*

(119) Kindly tell me the time of the free lectures at the Orthopædic Hospital and whether tickets are required. Also, I should be glad if you can tell me of any other lectures, free or otherwise, but not on massage or obstetrics.—*N. T.*

The lectures at the City Orthopædic Hospital, Hatton Garden, are at 4.30 P.M. on Friday afternoons, alternate weeks.

*South Africa.*

(120) Would you kindly tell me if it is any use for a trained monthly (not sick) nurse to go to Cape Colony in about a year's time? I have no friends to give me information.—*A. H.*

Unless you have sufficient money to keep yourself till you become known amongst doctors it would be foolish to go to South Africa.

Will you kindly tell me to whom I should write for particulars about going out to South Africa?—*A. M.*

Your question is too indefinite. Do you wish to join the Army Nursing Reserve? The address, in that case, is the Hon. Secretary, 18 Victoria Street, S.W. If you wish to go on your own account you will obtain the latest official returns from the Emigrants' Information Office, 31 Broadway, Westminster, S.W.

Will you kindly tell me what are the prospects of private nursing in South Africa?—*C.*

See reply to *A. H.* and *A. M.*

Will you kindly tell me the address of the Colonial Nursing Association? It is about South Africa that I wish to make inquiries.—*F. C. S.*

The Colonial Nursing Association, Imperial Institute, S.W.

*Massage.*

(121) Will you kindly tell us at what hospitals we could get fully trained in massage?—*Nurse C. and Nurse.*

At the National Hospital for the Paralysed and Epileptic, Queen's Square, Bloomsbury, W.C., and others.

Please tell me where I can learn massage in Paris?—*A. J.*

You had better apply to the matron, Hertford British Hospital, Rue de Villiers-Levallois-Perret, Paris.

I am a trained nurse and wish to study massage. As I cannot go to London, will you tell me of a school or hospital in Manchester where I could be taught?—*F. A.*

Apply to the secretary, The Society of Trained Masseuses, 12 Buckingham Street, Strand, W.C.

**Standard Books of Reference.**

"The Nursing Profession: How and Where to Train." 2s. net; post free 2s. 4d.
"Burdett's Official Nursing Directory." 3s. net; post free, 3s. 4d.
"Burdett's Hospitals and Charities." 5s.
"The Nurses' Dictionary of Medical Terms." 2s.
"Burdett's Series of Nursing Text-Books." 1s. each.
"A Handbook for Nurses." (Illustrated). 5s.
"Nursing: Its Theory and Practice." New Edition. 3s. 6d.
"Helps in Sickness and to Health." Fifteenth Thousand. 5s.
"The Physiological Feeding of Infants." 1s.
"The Physiological Nursery Chart." 1s.; post free, 1s. 3d.
"Hospital Expenditure: The Commissariat." 2s. 6d.

All these are published by the Scientific Press, Ltd., and may be obtained through any bookseller or direct from the publishers 28 and 29 Southampton Street, London, W.C.

### Travel Notes.

By Our Travelling Correspondent.

### LXXXVIII.—In Search of Sunshine.

Once more the season has come when many are thinking of wintering abroad, either in search of health, urgently in need of re-establishment, or simply because, being among the ranks of those vaguely spoken of as "delicate," a more sunny and cheerful climate is desirable, or, last of all, because, being free and untrammelled, they very wisely think to enjoy warmth, sunshine, freedom from fogs and frosts combined with an acquaintance, perhaps, with the vast art stores to be found on the Continent.

### Prosy Repetitions.

To our habitual readers I fear this paper will prove wearisome, but there are many new subscribers who have not listened to my winter warning voice before, so I must crave the indulgence of the former and beg them to excuse the serving up again of old dishes.

### If Delicate, Consult a Doctor.

I believe much harm is done by rashly choosing for oneself a winter residence without the help of a medical man. Mrs. A hears that Mrs. B's son has greatly benefited by a winter at Davos Platz ; therefore, though the youth was consumptive, and Mrs. A's daughter is feeble after a severe attack of gastritis, she reasons that a stay at Davos must be good, and accordingly drags off the girl to that sunny place to find that she is too weak to walk or get about much, and that there are no artistic or literary resources to while away the long days of convalescence. Always consult your doctor and abide by what he says ; many consent to ask advice, but only the strong-minded act upon it I notice.

### Clothing Required.

It is a fatal error, but, alas! a common one, to underrate the very real cold experienced in Italy or Southern France in the winter. Rome and Florence and even Nice and Mentone can be cruelly cold, quite as cold as England, only the brilliancy of the sun and the absence of damp, makes it agreeable and exhilarating; warm clothes, however, are essential. All underwear should be woollen, even for warm spring days. Light woollen combinations, high to the throat, and long to the wrists, should be worn, and the difference for warm and cold days should be in the outside garments. Thus safety is secured, and the dangers from the mistral and from alternately walking in brilliantly hot sun and in deep, cold shadow are avoided. Tailor-made coats and skirts, with white serge for smart wear, will furnish all you need for the day, and smart blouses make a pleasant change for the evening. Much dress is not good taste in an hotel. Have really comfortable boots and shoes, one pair of boots (not too thick) and two pairs of Oxford shoes, with smart indoor slippers, will be needed, and merino stockings. Avoid cotton or Lisle thread.

Do not stint yourself in wraps. A thick golf cape is a necessity, a lighter one of the same sort, and a waterproof, whilst a dust cloak proves very useful. One thick shawl too, is convenient for excursions and to supplement insufficient bed clothes. Use flannel nightgowns as a safeguard against damp beds on journeys.

### The Indispensable Tea-Basket.

To proud possessors of the real, very expensive, and terribly heavy article, I heed say nothing, but to humbler folk my advice is—make one. In the *Nursing Mirror* for August 26th, 1899, I gave full directions for constructing one exactly similar to the treasured friend which has travelled thousands of miles with me and yielded up its valued contents on the tops of icy peaks in Switzerland, in arid plains in Spain, amongst Roman ruins in Italy, on humble canal barges in Holland, and in the green and pleasant valleys of the Ardennes and the Moselle country.

What a comfort on long railway journeys. Tea is seldom met with, and milk is a luxury not to be thought of. How you are beloved of fellow-passengers! I have known this silent auxiliary to break down entirely the haughty reserve of compatriots — even the icy reserve of the British matron in charge of young and pretty daughters has been seen to thaw under its genial influence and to exchange cards! Think of it ; can English reserve unbend further?

### Luggage.

For this one pays heavily, so that circumspection in packing is needed. One large box will take all that is needed for several months and the cost of its transit to Central Italy will be about £1. The tendency is to take more of everything than one needs. Even now I find myself cumbered with abhorrent articles either for wear or domestic use ; these I hate and loathe as I conscientiously drag them from Dan to Beersheba, useless and cumbersome and am often tempted to leave them behind as the unregenerate mind counsels.

The best kind of trunk is a dome-shaped basket make, covered with leather, the tray takes one's hats well.

For sketching I have a few ideas peculiar to myself that I shall be pleased to impart to any fellow enthusiast.

### Avoid Heavy Handbags.

For many years my elegant dressing-bag fitted with everything that the heart of woman can desire in silver and crystal has been consigned to honourable retirement in a cupboard when I start continental wanderings, and its place taken by a vulgar rush bag, such as everyone knows, price 3s. 9d., size 16 inches wide by 12 inches deep. This I secure with a padlock, and line with pink jaconet, rendering it watertight. The whole weight being only 8½ oz. A bag this size will take everything needful for a night, including a hot-water bottle, which no traveller should be without.

### Necessary Comforts to be Remembered.

Take all the soap you will need, also a few packets of Hudson's powder, for cleaning your tea-things, etc., and for occasional descents upon bedroom pails, that often, even in good hotels, cry aloud for attention. You will, of course, take a little brandy, but a store is not necessary, as it is good abroad and not dear.

A good supply of tooth-powder is wanted, and a little castor oil, and perhaps a bottle of Eno's fruit salt.

Take a common 6d. tin kettle, one of the kind whose handle tucks down—it will be a thing of beauty and a joy for ever to you. The kettle of France and Italy is a vast iron or copper receptacle holding some gallons and of untold weight. A little saucepan of the same kind as the kettle will be useful for heating milk, etc., and when you are settled in your temporary home invest in the tin jug of the country, with a basket handle. They live continually among the hot embers of the wood fires, and so in the winter one has warm water in season and out of season.

### TRAVEL NOTES AND QUERIES.

Rules in Regard to Correspondence for this Section.—All questioners must use a pseudonym for publication, but the communication must also bear the writer's own name and address as well, which will be regarded as confidential. All such communications to be addressed "Travel Editor, 'Nursing Section of *The Hospital*,' 28 Southampton Street, Strand." No charge will be made for inserting and answering questions in the inquiry column, and all will be answered in rotation as space permits. If an answer by letter is required, a stamped and addressed envelope must be enclosed, together with 2s. 6d., which fee will be devoted to the objects of "The Hospital" Convalescent Fund. Any inquiries reaching the office after Monday cannot be answered in "The Hospital" of the current week.

